# Crushing the curve, the role of national and international institutions and policy makers in COVID-19 pandemic

**DOI:** 10.3906/sag-2004-167

**Published:** 2020-04-21

**Authors:** Zeliha KOÇAK TUFAN, Bircan KAYAASLAN

**Affiliations:** 1 Department of Infectious Diseases and Clinical Microbiology, Faculty of Medicine, Ankara Yıldırım Beyazıt University, Ankara Turkey; 2 Executive Board Member of Council of Higher Education of Turkey (YÖK); 3 Member of COVID-19 Advisory Committee of Ministry of Health of Turkey

**Keywords:** Pandemic, coronavirus, SARS-CoV-2, World Health Organization, CDC, Ministry of Health

## Abstract

Nobody can be fully prepared to a pandemic. Of course there are signs of it, the scientists can predict, alarming speeches can be made. But there are always alarmist people around, maybe that is why sometimes even the most serious warnings may be not considered by the authorities on time. The first patients may be lost without a proper diagnosis. When everybody realizes that there may be a big problem in the horizon, sometimes it is too late. That is why it is very important to monitor contagious diseases and follow the warnings and releases of national and international disease control centers and other related organizations. China celebrated Lunar New Year with more than 40 thousand families on the 18 of January 2020. Nobody seem to be expecting this emerging new viral pneumonia outbreak appeared in Wuhan, in the last days of 2019, will break the chains and turn out to be a pandemic! But maybe this time it was not too late. There were four important pandemics within the last century: Spanish Flu, Hong Kong Flu, Asian Flu and Swine Flu. Each left different story behind. Millions of people had infected, hundreds, thousands of people died. This time, the Modern World had different tools to limit the SARS CoV2 outbreak. The national and international institutions of our globe were all communicating and taking precautions in a very fast manner than ever. However, this time, unexpectedly, the SARS-CoV-2 contagion was also faster. Besides the international organizations like WHO, UNESCO and UNICEF, the roles of local authorities, health ministries, disease control centers, health protection agencies, research centers and universities are all very important in different operational levels to control and survive from the pandemic. This paper will review the immediate response of different national and international institutions and authorities to COVID-19 pandemic.

## 1. Introduction

On the last day of 2019, pneumonia cases with unknown etiology from Wuhan City, Hubei Province was reported to China’s World Health Organization’s [WHO] Country Office. The number of the cases increased, and the source was thought to be related with the seafood market in Wuhan city. The causative agent was isolated on January 7, 2020 and identified as a new type coronavirus [1]. After one month had passed from the first cases of the 2019-nCoV outbreak, later named as SARS CoV2, WHO declared the outbreak as a ‘*Public Health Emergency of International Concern’ *and provided advice to the global community to control the outbreak on January 30. Almost more than one month later, it was declared as a controllable pandemic on March10^1^1World Health Organization (2020). Statement on the second meeting of the International Health Regulations (2005) Emergency Committee regarding the outbreak of novel coronavirus (2019-nCoV) [online]. Website https://www.who.int/dg/speeches/detail/who-director-general-s-opening-remarks-at-the-mission-briefing-on-covid-19---12-march-2020 [accessed 05 April 2020]..

Nobody can be fully prepared to a pandemic. Of course, there are signs of it, the scientists can predict, alarming speeches can be made, papers can be written. But there are always alarmist people around, maybe that is why sometimes even the most serious warnings may not be heard by the authorities on time. The first patients may be lost without a proper diagnosis. When everybody realizes there may be a big problem in the horizon, sometimes it is too late. That is why it is very important to monitor contagious diseases and follow the warnings and releases of national and international disease control centers and other related organizations. But what happens if they are also late? In terms of COVID-19, the first cases were reported from Wuhan on the last day of 2019. People were celebrating new year all over the world. First the new disease attracts attention of the scientists, medical world, then media and hall world. The number of the cases increased dramatically, and the speed of the curve became high. Still, many believed that it would be a limited outbreak^2^2The Star, Star Media Group (2020). China celebrated Lunar New Year with more than 40 thousand families on the 18th of January 2020 [online]. Website https://www.thestar.com.my/news/regional/2020/02/06/wuhan-neighbourhood-sees-infections-after-40000-families-gather-for-potluck [accessed 05 April 2020].. Nobody seemed to expect this new viral pneumonia outbreak emerging in Wuhan to break the chains and turn out to be a pandemic. 

There were four important pandemics within the last century: Spanish Flu, Hong Kong Flu, Asian Flu and Swine Flu. Each left different story behind. Millions of people had infected, hundreds, thousands of people died^3^3Centers for Disease Control and Prevention (2020). Past pandemics [online]. Website https://www.cdc.gov/flu/pandemic-resources/basics/past-pandemics.html [accessed 05 April 2020].. In none of them, social media was the witness. Currently, hundreds and thousands of videos and photos have been shared in the social media, some of them are misleading and nothing to do with current outbreak. People have been searching more and more every day to reach true, updated data, as if people around the world were starving knowledge about the current COVID-19 outbreak.

In the modern world, travel and communication opportunities seem to be limitless and fast, so is the spread of SARS-CoV-2. This time, the modern world had different tools to limit the SARS-CoV-2 outbreak. Modern laboratories were ready to investigate the new virus, produce new diagnostic tools, modern hospitals were ready to accept patients. National and international authorities were communicating with each other. Technology was on our side. So, the 2020 pandemic could be controlled easily more than ever. But is that what was happened? In the modern world, people have different preferences. They are less eager to obey the rules, they feel themselves more free or need more freedom, which overall can crush the quarantine procedures easily. Everybody can seek the top medicines, even if it is on the way in a trial. Patients can be more anxious. 

Besides the international organizations like WHO, UNESCO and UNICEF, the roles of local authorities, health ministries, other ministries, disease control centers, health protection agencies, research centers and universities are all very important in different operational levels to control and survive from the pandemic. This paper will review the immediate response of different national and international institutions to COVID-19 pandemic. 

## 2. World Health Organization (WHO)

From 1948 till now WHO has been working on to *promote health, keep the world safe, and serve vulnerable people*. There are 150 WHO country offices. More than 7000 people from more than 150 countries are working under WHO. Beside many, one of their goal is also to *improve monitoring, data and information*. As WHO stated in its website, *they analyses data, provide advice, coordinate with partners, help countries prepare, increase supplies and manage expert networks*^4^4World Health Organization (2020). Who we are [online]. Website https://www.who.int/about/who-we-are [accessed 13 April 2020].*.*That is why WHO was the center of the news from the first time on.

The different roles of WHO in COVID-19 outbreak are extremely important. Mainly the most important role at the very beginning of outbreak was the *informative role* of WHO, of course followed by *preparedness and response* acts. The interim name of the virus and the disease was also recommended by WHO as “2019-nCoV acute respiratory disease” which was named as COVID-19 (Co: corona, VI: virus, D: disease, which first cases appear in 2019) later on February 11 provided by International Classification of Diseases (ICD)^5^5World Health Organization (2020). Novel Coronavirus (2019-nCoV) Situation Report - 10 [online]. Website https://www.who.int/docs/default-source/coronaviruse/situation-reports/20200130-sitrep-10-ncov.pdf?sfvrsn=d0b2e480_2 [accessed 30 January 2020].. They stated that further international spread of cases might appear in any country and every country should be prepared for containment. WHO declared the outbreak as a *Public Health Emergency of International Concern* and gave advice to the global community to control the outbreak on January 30 and more than one month later, on March 10, defined outbreak as a controllable pandemic^6^6World Health Organization (2020). Coronavirus disease (COVID-19) outbreak [online]. https://www.who.int/westernpacific/ emergencies/covid-19 [accessed 13 April 2020]..

WHO published the Situation report-1 on January 20 when the total confirmed case number had reached 282 and four countries, China, Thailand, Japan and Korea, had reported 2019-nCoV cases. Although closely monitored, the situation had been not expected to cause a pandemic on the early days of January and no restrictions for travel or trade were advised^7^7World Health Organization (2020). WHO Statement regarding cluster of pneumonia cases in Wuhan, China [online]. Website https://www.who.int/china/news/detail/09-01-2020-who-statement-regarding-cluster-of-pneumonia-cases-in-wuhan-china [accessed 9 January 2020].. Later, when the case numbers had been dramatically increasing, *WHO assessed the risk of this event to be very high in China, high at the regional level and high at the global level*. On January 23, the situation was evaluated by WHO-International Health Regulations Emergency Committee and stated that *it’s was bit too early to consider this event as a Public Health Emergency of International Concern*^8^8World Health Organization (2020). Novel Coronavirus (2019-nCoV) Situation Report - 3 [online]. Website https://www.who.int/docs/default-source/coronaviruse/situation-reports/20200123-sitrep-3-2019-ncov.pdf?sfvrsn=d6d23643_8 [accessed 23 January 2020]. .

The situation reports of WHO have been followed by millions. The situation reports provide situation updates, surveillance reports, advices for preparedness and response, as well as country responses, subjects in focus and strategic objectives, all day by day^9^9 World Health Organization (2020). Novel Coronavirus (2019-nCoV) Situation Report - 1 [online]. Website https://www.who.int/docs/default-source/coronaviruse/situation-reports/20200121-sitrep-1-2019-ncov.pdf [accessed 20 January 2020]. [2,3]. In the modern world where millions of reports and news are releasing daily, these situation reports are very informative and brief to follow up. 

Besides situation reports, WHO also provide information under different topics: media resources, advice for public, travel advice, technical guidance, training and exercises. All are very important to reach correct and updated information during a global outbreak. Training and exercises section provides online COVID-19 training and simulation exercises plus simulation packages in several languages. From the treatment facility design to infection prevention and control measures, a broad range of online training are provided, which are extremely important, especially for those countries which lack enough professionals in the area^10^10World Health Organization (2020). Coronavirus disease 2019 [online]. Website https://www.who.int/emergencies/diseases/novel-coronavirus-2019 [accessed 10 April 2020]..

WHO announced Donor Alert on the February 4, 2020 when the risk assessment was as *very high* for China, just *high* at the global level. The Global Resource Requirement was stated to be USD 675.5 million, of which USD 61.5 million were for the WHO, for urgent preparedness and response activities, primarily to help protecting states with weaker health systems. The Global Strategic Preparedness and Response Plan includes establishing international coordination and operational support, country readiness and response operations, accelerating priority research and innovation. As of April 9 2020, over USD 356 million was already received by WHO^11^11World Health Organization (2020). Novel Coronavirus (2019-nCoV) Donor Alert [online]. Website https://www.who.int/docs/default-source/coronaviruse/donor-alert.pdf [accessed 4 February 2020]..

## 3. Disease control and prevention centers and health protection agencies

Many countries have active surveillance, protection and control systems for communicable or contagious diseases. So, either they have centers for diseases control and prevention or health protection agencies. 

CDC of USA is among the most popular one. The mission of CDC is *to protect America from health, safety and security threats, both foreign and in the U.S*.^12^12CDC (2019). About CDC 7-24 Mission, Role and Pledge [online]. Website https://www.cdc.gov/about/organization/mission.htm [accessed 13 May 2019].. CDC’s Traveler Health web site is also providing brief information on ongoing outbreaks and illnesses as well as protection advices for those who want to travel to any country. This web site is being updated continually and one can also find a clinic in a destination or access help from it. Information can be found both for physicians and others. In case of a pandemic the CDC web site is very informative especially for those countries lacking health professionals and advisory committees^13^13CDC (2020). Travelers Health [online]. Website https://wwwnc.cdc.gov/travel/destinations/list/ [accessed 10 April 2020]..

As an immediate response to COVID-19 pandemic CDC provided useful tools for both public and health professionals. *How to protect yourself, what to do if you are sick* are the immediate titles when you open their website, which are the most important topics for the public in an outbreak. To cope with the pandemic, keeping home environment safe and cleaning, keeping healthy life were all among the useful advices for the public. Extra precautions and a self-checker guide are also provided^14^14CDC (2020). Coronavirus (COVİD-19) [online]. Website https://www.cdc.gov/coronavirus/2019-nCoV/index.html [accessed 10 April 2020].. When the number of COVID-19 cases increased dramatically, CDC advised that the surgical masks should be kept for medical staff and they provided brochures and videos on how to make cloth face covering for the public^15^15CDC (2020). Use of Cloth Face Coverings to Help Slow the Spread of COVID-19 [online]. Website https://www.cdc.gov/coronavirus/2019-ncov/downloads/DIY-cloth-face-covering-instructions.pdf [accessed 10 April 2020].. The US Surgeon General Dr. Jerome Adams can be seen on a video to show how to prepare homemade masks^16^16CDC (2020). Coronavirus disease 2019 (COVİD-19) Recommendation Regarding the Use of Cloth Face Coverings, Especially in Areas of Significant Community-Based Transmission [online]. Website https://www.cdc.gov/coronavirus/2019-ncov/prevent-getting-sick/cloth-face-cover.html [accessed 3 April 2020].. Still, there were also reactions from many US citizens criticizing lack of masks in an outbreak in a country which its economic is the number one in the world. 

For health care professionals CDC also provide brief and useful info under these titles^17^17CDC (2020). Coronavirus disease 2019 (COVİD 19) For Healthcare Professionals [online]. Website https://www.cdc.gov/coronavirus/2019-nCoV/hcp/index.html [accessed 7 April 2020].:

· Evaluating and testing

· Clinical care guidance

· Infection control

· Optimize personal protective equipment supply

· Potential exposure at work

· First responder guidance 

· Guidance for non-US facilities

· Guidance for ambulatory care setting, 

· Guidance for pharmacies 

· Guidance for dental settings

Of course, giving professional guidance for population and health care professionals are extremely important in an outbreak to prevent chaos. The panic level of the population is usually high in a pandemic so controlling the false and misguided information in social media is not easy. Having a trustworthy information source is very important. Outbreaks cause panic not only in the public but also among the professionals as well. The infection control teams and infectious disease [ID] physicians may be exhausted during the pandemic; all the care givers should know at least the basics of infection control. A physician may be a very good nephrologist, but they need what to do in their dialysis facilities in an outbreak for infection control. Providing brief information about using personal protective equipment (PPE) and hand hygiene as well as sharing the updated epidemiological features of the disease are all extremely useful^18^18CDC (2020). Coronavirus disease 2019 (COVİD 19) For Healthcare Professionals [online]. Website https://www.cdc.gov/coronavirus/2019-nCoV/hcp/index.html Access date: 7 April 2020..

In Turkey, the Public Health General Directorate published guidelines for current pandemic for both public and health care professionals, with the help of COVID-19 Advisory Committee, and updated all the relevant data day by day [2,3].

## 4. Ministry of healths and parliements 

Ministry of health of countries are in the core of the organizations during a pandemic. Different country’s health ministries and parliaments response to pandemic was reviewed here (provided information in detail in Tables 1 and 2)^19^19Oxford Covid-19 Government Response Tracker [Online]. Website https://www.bsg.ox.ac.uk/research/research-projects/oxford-covid-19-government-response-tracker. Access date: 12 April 2020.

**Table 1:** Goverments response to COVID-19 pandemic by date [17].

Country / Preventing measure	China	France	Germany	Italy	Japan	Singapore	S.Korea	Spain	Turkey	UK	US	Firstapplied
School closing	26-Jan	16-Mar	16-Mar	23-Feb		8-Apr	3-Feb	9-Mar	16-Mar	23-Mar	5-Mar	26-Jan
Workplace closing	26-Jan	17-Mar		23-Feb		7-Apr	22-Mar	14-Mar		21-Mar	19-Mar	26-Jan
Cancel public events	23-Jan	29-Feb	10-Mar	23-Feb		13-Mar	31-Jan	10-Mar	16-Mar	21-Mar	12-Mar	23-Jan
Close public transport	23-Jan	16-Mar							24-Mar			23-Jan
Public information campaigns	20-Jan	24-Jan	27-Feb	31-Jan	4-Feb	2-Jan	20-Jan	31-Jan	7-Feb	31-Jan	17-Mar	2-Jan
Restrictions on internal movement	23-Jan	17-Mar	20-Mar	21-Feb			23-Feb	14-Mar	15-Mar	23-Mar	19-Mar	23-Jan
Country’s first response	20-Jan	24-Jan	27-Feb	31-Jan	4-Feb	2-Jan	20-Jan	31-Jan	7-Feb	31-Jan	5-Mar	

**Table 2:** COVID-19 pandemic, government response and preventive measure by date [17].

Preventing Measures	China	France	Germany	Italy	Japan	Singapore	S. Korea	Spain	Turkey	UK	US
International travel controls											
Screening		23-Jan	28-Feb	23-Jan	7-Jan	2-Jan			24-Jan		
Quarantine on high-risk regions	25-Feb	31-Jan			9-Mar						2-Feb
Ban on high-risk regions	26-Mar	18-Mar	16-Mar	30-Jan	3-Apr	23-Jan	3-Feb	10-Mar	5-Feb		2-Mar
Contact tracing											
Limited contact tracing – not done for all cases	1-Jan	26-Feb	18-Mar		15-Jan		29-Jan	31-Jan	14-Jan
	
Comprehensive contact tracing – done for all cases	23-Jan		22-Jan	31-Jan		23-Jan	11-Feb			31-Jan	
Testing framework											
Only testing those who both (a) have symptoms, and (b) meet specific criteria	1-Jan	17-Mar	27-Jan	31-Jan	15-Jan		21-Jan	24-Jan		20-Jan	28-Feb
Testing of anyone showing COVID19 symptoms				26-Feb		23-Jan			14-Jan		4-Mar

### 4.1. Turkey

Turkey immediately implemented numerous preventive measures to combat with spread of COVID-19 infection since onset of the pandemic (Table 3). Turkey set up the Scientific Advisory Board the COVID-19 Advisory Committee, in the early time of on January10, 2020, before WHO accepted it as a pandemic in mid-March, following the emergence of the coronavirus outbreak. Board consists of different branch specialist including infectious disease and clinical microbiology, respiratory system, clinical microbiology, emergency disease, epidemiology, pediatric infection, virology, and public health and internal medicine. 

**Table 3:** Timeline of COVID-19 and major implementations in Turkey.

31 December 2019	Pneumonia cases with unknown etiology were reported from Wuhan.
1 January 2020	Huanan Seafood Market in Wuhan was closed.
6 January 2020	Operations Center for COVID-19 was established by the General Directorate of Public Health in Ministry of Health.
7 January 2020	Novel coronavirus was declared as the cause of the outbreak
10 January 2020	Coronavirus Science Board, The COVID-19 Advisory Committee of Turkey convened and held its first meeting.
14 January2020	The 2019-nCoV Disease Guideline was published by the suggestions of the Scientific Board
30 January 2020	WHO declared the outbreak as a “Public Health Emergency of International Concern”.Turkish Airlines cancelled flights to and from China.
1 February 2020	Turkish citizens were evacuated from China and isolated for 14 days
2 February 2020	Turkey cancelled all flights to China and started to screen all passengers for the fever and respiratory symptom
23 February	2020 Turkey closed Turkey-Iran border gates with Iran and banned flights to and from Iran.
10 March 2020	The first confirmed COVID-19 case was reported.
12 March 2020	Turkey closed schools and universities starting from 16 March.
16 March 2020	Turkey banned in-person visits and family interviews in prisons.
24 March 2020	Turkey restricted going out and traveling by public transport of people who are older than 65 years and people with immune system deficiency or chronic disease.
3 April 2020	Turkey implemented the curfew for people under the age of 20
9 April 2020	Turkey declared a curfew that applies to all citizens except for health-care workers and security workers for weekends

The Board has convened regularly, and published guideline named COVID-19 (SARS-CoV-2 Infection) Guide, and updated this guide according to the scientific data. This guide contains the topics of general information about SARS-CoV-2 and COVID-19 infection, management of COVID-19 infection, management of emergency patients with COVID-19, management of patients with COVID-19 in outpatient clinics, management of patients with severe COVID-19 infection, management of healthcare worker who exposure to COVID-19 and screening in contact person [3]. 

The first confirmed COVID-19 case was reported on March 10, 2020. The first death due to COVID-19 in the country was declared on March 17 [3]. Turkish government has adopted several containment measures, including social distancing, travel bans on visitors from high-risk countries and quarantine for nationals returning from those countries, and the closures of schools, stores, and entertainment venues. On March 12, the government declared that all of the schools and universities would be closed starting from March 16. Turkey suspended in-person visits and family interviews to prevent the spreading of coronavirus in prisons for two weeks on March16 and this period was prolonged during the pandemic. On March 30, the personnel such as execution protection officers working in criminal execution institutions and having the possibility of contact with prisoners have been decided to work in the form of 14-day shifts if possible, or at least 7-day shifts. It closed airline to flights from high-risk country firstly China, then Iran and Italy and closed to Turkey-Iran border gates to passenger entrances. Firstly, Turkey banned flights to and from China. Passengers were screened at airports for the fever and respiratory symptoms. And this implementation extended to the passengers coming from the other countries 2020T.C. Sağlık Bakanlığı (2020). COVID-19 (Yeni Koronavirüs Hastalığı) Bilimsel Danışma Kurulu Kararları,Kurumlar İçin Alınan Kararlar [online]. Website https://covid19bilgi.saglik.gov.tr/tr/alinan-karalar.html [accessed 13 April 2020].

Turkey implemented some restriction for the decreasing of people movement. Turkey parliament restricted going out for people 65 years old and older and citizens who have immune system deficiency, chronic lung disease, asthma, COPD, chronic cardiovascular disease, chronic renal disease, hypertension and chronic liver disease, and citizens who use drugs that disrupt the immune system. Turkey started implementation of curfew restricting members of public under 20 years old (born after January 1, 2000) from leaving their homes unless precisely necessary from 3rd of. Entry and leave by private vehicles to 31 big provinces including Istanbul that has one-fifth of Turkey population was banned. The wearing of facemasks in crowded areas including stores and mass transportation became mandatory April 3, 2020. On April 9, Turkey declared curfew for all citizens except for health-care workers and security workers for weekends^21^21Wikipedia (2020).Timeline of the 2020 coronavirus pandemic in Turkey [online]. Website https://en.wikipedia.org/wiki/Timeline_of_the_2020_coronavirus_pandemic_in_Turkey [accessed 13 April 2020]..

### 4.2. Italy

Italy has become the new epicenter of the COVID-19 pandemic in Europe with the fastest increasing rates. After the first two confirmed cases reported on January 31, more than 150,000 people became infected within 2 months and increase keeps growing^22^22Worldometer (2020). Total coronavirus cases in Italy [online]. Website https://www.worldometers.info/coronavirus/country/italy/ [accessed 13 April 2020].. Italy has surpassed China as the country with the highest death number in a short time. Lombardy, the northern region at the center of the outbreak, had been lockdown^23^23Jason Horowitz, Emma Bubola and Elisabetta Povoledo. Italy, Pandemic’s New Epicenter, Has Lessons for the World [online]. Website https://www.nytimes.com/2020/03/21/world/europe/italy-coronavirus-center-lessons.html [accessed 3 March 2020]..

Italy suspended the flight to and from China after the first confirmed cases on January 31, and declared a national emergency. The center of Italian cases has been identified as northern Italy and firstly eleven municipalities in northern Italy, then the entire Lombardy and other northern provinces and finally the whole Italy under quarantined^24^24Wikipedia (2020). Coronavirus pandemic in Italy [online]. Website https://en.wikipedia.org/wiki/2020_coronavirus_pandemic_in_Italy [accessed 13 April 2020].. Italy implemented a series of restrictions of increasing severity. The government firstly divided the Italian national territory into three areas on March 1;

· Red zones (where the whole population was in quarantine),

· Yellow zones (where schools, theatres, clubs and cinemas were closed, social and sport events were suspended), and 

· Green area (the rest of national territory where safety and prevention measures are advertised in public places). 

With the increasing number of confirmed and fatal cases, preventive measures have been expanded to the whole country. All of schools, universities, museums, cinemas, theatres, and any other social or cultural centers were closed, and all sporting activities were cancelled on March 9, and all restaurants and bars are closed on March 11. Most shops excluding supermarkets and pharmacies were also closed^25^25Wikipedia (2020). Coronavirus pandemic in Italy [online]. Website https://en.wikipedia.org/wiki/2020_coronavirus_pandemic_in_Italy [accessed 13 April 2020].[4].

### 4.3. United Kingdom (UK)

The first two confirmed COVID-19 patients were declared on January 29 and transmission within the UK was confirmed in February. The number of COVID-19 cases increased rapidly in March. In response, the government has implemented a range of measures. It raised the risk level from COVID-19 from ‘moderate’ to ‘high’ on 12 March and one day later postponed all sporting events. UK announced citizens to work from home and avoid pubs and restaurants to give the NHS time to deal with the pandemic on 20 March. The UK government closed most school across the country on 23 March. The citizens were banned to go outside except for buying foods and exercising once a day or going to work if they absolutely had to in the same day. 

In March, hospitals in UK have begun to cancel all nonurgent elective operations. They announced also to stay at home for those who have symptoms of COVID-19 and not to visit a GP, pharmacy or a hospital and to use a dedicated online self-assessment, nonemergency medical helpline. The National Health Service emptied 30,000 hospital beds by postponing treatment of nonurgent patients and supplied additional use of 20,000 beds in private sector facilities. The government requested retired NHS staff to return to work for combat the pandemic and more than 20,000 former the National Health Service [NHS] staff returned to work in a less than two weeks. An agreement was achieved with almost the entire private health systems to overcome the pandemic on 21 March, and 20,000 medical staff started working for national purposes, 20,000 private sector beds were added to national sources. Up to 30,000 hospital beds were also provided by delaying nonemergency patients and discharging the patients in good health. Additionally, temporary critical care hospitals were established to overcome of bed shortage^26^26Wikipedia (2020). Coronavirus pandemic in the United Kingdom [online]. Website https://en.wikipedia.org/wiki/2020_coronavirus_pandemic_in_the_United_Kingdom#National_Health_Service [accessed 13 April 2020]..

## 4.4 Spain

Spain is one of the most affected country by the COVID-19. The first confirmed patient was reported on 31th of January and many cases were detected by 24th February, origin of these cases was an Italian medical doctor who visited Spain, and then the disease spread to other communities. Within two months, Spain became the country most affected by the outbreak of COVID-19. It has the highest total number of cases and second highest number of deaths in Europe. Even the Spain Ministry of Health statistics is claimed to be inaccurate and incomplete. It was stated that the actual numbers were much more than the determined numbers because the tests were performed only for severe patients and not performed for patients with mild course, as well as there are older fatal cases on nursing home who might die before being tested.

Spain implemented some preventive measures. The first cancellation of all educational levels was announced by the regional community government of Madrid on March 9 and in advanced time, the other regional governments cancelled all educational activities. On March 13, a national state of alarm was declared, and lockdown started to be implemented on March 15 for 15 days and then extended until April 11. All trips were banned, all residents were mandated to stay at home except for emergency and purchasing of food and drugs. Nonessential shops and businesses had been closed since March 30. On March 28, all nonessential activities were banned by the Spanish government and workers were allowed with leave paid. 

In the last decade, as the economy spiraled downward in Spain, heavy spending cuts were being implemented. Investment in health sector expenditure has dropped far below the current average level of European Union. Due to hospitals were privatized, it has become harder to overcome the epidemic and coordinate the crisis. Before the epidemic, there were unemployed doctors or emigrated doctors to look for work, during the pandemic, retired healthcare workers were called back to the job and medical students were recruited to perform some tasks. 

Spain, like other many countries, suffered from shortage of intensive care beds. Although Spain increased the capacity of hospital beds to triple until the end of March, intensive care units were full over 80% with patients suffering from COVID-19. In Spain, about 10% of the cases are among healthcare workers. Insufficiency in personnel protective equipment is blamed for causing a high proportion of infection among healthcare workers. Twelve nurses and doctors have died due to the illness^27^27Wikipedia (2020). Coronavirus pandemic in Spain [online]. Website https://en.wikipedia.org/wiki/2020_coronavirus_pandemic_in_Spain [accessed 13 April 2020]..

### 4.5. Singapore

Singapore is one of the countries that fight well against the epidemic. The first confirmed case, a Chinese national coming from Wuhan, was declared on January 23. Although the first case was detected in the very first month of the epidemic, the total number of reported cases so far (April 11) is 3252 and total number of deaths is only 10^28^28Worldometer (2020). Total coronavirus cases in Singapore [online]. Website https://www.worldometers.info/coronavirus/country/singapore/ [accessed 13 April 2020]..

Firstly, passengers coming from Wuhan was started to be checked for high temperature on 2 January and this screening was expanded to all passengers coming from China on 20 January and individuals with pneumonia were isolated for 14 days and quarantine was extended to all passenger coming from Wuhan. Singapore advised citizens to avoid non-essential travel to China on January 27. On 31 January, entry to Singapore, or to transit through Singapore was banned for all passengers with recent travel history to mainland China within the last 14 days. Stay-home notice was implemented for all Singapore residents and passengers coming from China. On March 4 visitors arriving from South Korea, Iran and northern Italy were banned to entry country. In the advanced time of epidemic, entry ban extended to visitors coming from Italy, Japan, France, Spain and Germany. The passengers with fever and respiratory symptoms were screened with swab tests. Any traveler showing symptoms at checkpoints were isolated for 14 days, even with negative results for COVID-19. Collective activities like cultural, sports and entertainment events with 250 people or more were deferred or cancelled on March 13. In prison, family interviews were suspended between April 7 and May 4. Visitors were banned from visiting relatives in the hospital unless necessary on April 7^29^29Ministry of Health Singapore (2020). Extension of Precautionary Measures to Minimize Risk of Community Spread in Singapore [online]. Website u29f9 [accessed 13 April 2020].,^30^30Wikipedia (2020). 2020 coronavirus pandemic in Singapore [online]. Website https://en.wikipedia.org/wiki/2020_coronavirus_ pandemic_in_Singapore [accessed 13 April 2020]..

### 4.6. China 

Mysterious pandemic firstly started in Wuhan, a city of Hubei Province in China at the end of 2019. China closed Huanan Seafood Wholesale Market, the suspected source of the initial pneumonia cases, on January 1, 2020. The CDC China isolated the first novel coronavirus strain on January 7. The first death because of the virus occurred on January 9^31^31Wikipedia (2020). Timeline of the 2019–20 coronavirus pandemic from November 2019 to January 2020 [online]. Website https://en.wikipedia.org/wiki/Timeline_of_the_2019–20_coronavirus_pandemic_from_November_2019_to_January_2020#Events,_reactions,_and_measures_in_mainland_China [accessed 13 April 2020]..

China rolled out an aggressive disease control effort. In the first stage of outbreak, a general infection prevention measures that promoted universal temperature monitoring, masking, and hand washing was implemented. As time goes and scientific data are collected, it was focused on improving key performance indicators like enhancing the speed of case detection, isolation of patients and their close contacts and early treatment. During the late phase of the outbreak, the primary focus point became reducing cluster of cases, by the way of the control of transportation capacity to reduce the movement people and cancelling of mass gathering^32^32World Health Organization (2020). Report of the WHO-China Joint Mission on Coronavirus Disease 2019 (COVID-19) 16-24 February 2020 [online]. Website https://covid19.21wecan.com/covid19en/c100037/202004/cbe307e7e76044159265e6da390b46d3/files/aa5094f0086344f6beba88dfab848a9a.pdf [accessed 28 February 2020]..

In the early stage, pandemic was not being able to under control by Wuhan local authorities, it spreads rapidly to other provinces and countries. On January 22, the government announced a quarantine, valid from January 23, and cancel all flights and trains from Wuhan. However, approximately 100,000 people had already departed from Wuhan on the same day by train and many of them succeeded bypassing the checkpoints by using antipyretics. Border gates were closed, and Wuhan city was under lockdown on January 23. The entire Hubei province came under a city-by-city quarantine on January 24^33^33Wikipedia (2020). Timeline of the 2019–20 coronavirus pandemic from November 2019 to January 2020 [online]. Website https://en.wikipedia.org/wiki/Timeline_of_the_2019–20_coronavirus_pandemic_from_November_2019_to_January_2020#Events,_reactions,_and_measures_in_mainland_China [accessed 13 April 2020].. The number of cases were shown to be highly associated with the population emigration from Wuhan to other cities of Hubei, and after lockdown, population migration was greatly inhibited, and spreads of virus were prevented [5]. 

China took some comprehensive, strict prevention and control measures to battle the epidemic. Hubei raised Class 2 Response to Public Health Emergency on 22 January and by 29 January, all parts of mainland raised to Class 1 Response, the highest response level. Provincial coordinated investigation into epidemic area and managed human movement. On 26 January, spring festival holiday was extended, and educational institutions postponed the start of school. Many sportive activities were postponed due to outbreak. The State General Administration of Sports declared to be suspended of all sporting events until April on 25 January^34^34Wikipedia (2020). 2019–20 coronavirus pandemic in mainland China [online]. Website https://en.wikipedia.org/wiki/2019%E2%80%9320_coronavirus_pandemic_in_mainland_China#Cancellations,_delays_and_shutdowns [accessed 13 April 2020].. In March, the government strictly restricted international travel and limited numbers of flights to the country and did not allow foreigners to enter the country^35^35Ministry of Foreign Affairs of the People’s Republic of China (2020). Ministry of Foreign Affairs of the People’s Republic of China, National Immigration Administration Announcement on the Temporary Suspension of Entry by Foreign Nationals Holding Valid Chinese Visas or Residence Permits [online]. Website https://www.fmprc.gov.cn/mfa_eng/wjbxw/t1761867.shtml. [accessed 13 April 2020].. China ordered a nationwide screening for the purpose of identification and immediate isolation of coronavirus-infected travelers at all departments of transportation including airports, railway stations, bus stations and port. All wildlife trade was banned with immediate effect^36^36Wikipedia (2020). 2019–20 coronavirus pandemic in mainland China[online]. Website https://en.wikipedia.org/wiki/2019%E2%80%9320_coronavirus_pandemic_in_mainland_China#Cancellations,_delays_and_shutdowns [accessed 13 April 2020]..

In a short time, due to the accumulation of a great number of patients in hospitals, the need for medical staff and hospital beds appeared in Wuhan, Hubei. China’s National Health Commission sent medical staff to Wuhan City to meet the need of healthcare personnel and to combat the novel coronavirus outbreak in the region. The government constructed two emergency specialty field hospitals to meet the need for hospital beds. China increased daily number of producing of facemask from 10 million (half of the world production) to 116 million to supply the increased consumption need. It used some high-tech products like delivery drones, artificial intelligence and facial recognition system^37^37Wikipedia (2020).Timeline of the 2019–20 coronavirus pandemic from November 2019 to January 2020 [online]. Website https://en.wikipedia.org/wiki/Timeline_of_the_2019–20_coronavirus_pandemic_from_November_2019_to_January_2020#Events,_reactions,_and_measures_in_mainland_China [accessed 13 April 2020]..

### 4.7. Germany

In the beginning of the outbreak, the government declared the spread of COVID-19 as a “very low health risk” for Germans. In the following days, government continued to defend low risk opinion. The first confirmed case was reported on January 28, and in the same day, after a suspected case in a private airplane, the company cancelled all flights to China. On February 13, the German Health Minister announced that direct flights between Germany and China would not cancelled. But after several weeks, the German Ministry of Transport announced to stop all flights from Iran and China on March 16. However, at the same dates flights from China and Iran continued due to bilateral agreements. Germany has a robust healthcare system to combat the outbreak. They have enough hospital beds to meet the increased needs. The major problem is having chronically inadequate medical staff. Medical students were used to help out in the most overwhelmed units.

On March 22, the government banned gatherings of more than two persons and required the condition that there was at least a minimum distance of 1.5 meters between people in public except for families, partners or people living in the same household. Restaurants and services like hairdressers were closed. Germanys closed schools and daycare centers to slow down the spread of the novel coronavirus on 13 March. In the second part of March, the doctors and other healthcare workers criticized shortage of PPE. Some big car manufacturers and banks donated mask for healthcare workers^38^38Wikipedia (2020). Coronavirus pandemic in Germany [online]. Website https://en.wikipedia.org/wiki/2020_coronavirus_pandemic_in_Germany#Government_reactions [accessed 13 April 2020]..

### 4.8. Japan

Japan was among the countries the first cases confirmed outside China, according to WHO announcement on January 20 [3]. On February 3, Japan banned passengers coming from Hubei Province or those who had a Chinese passport officially from Hubei and those who visited Hubei in the last 14 days to enter the country. On March 5, new quarantine restrictions were announced for all visitors coming from China and South Korea.

A cruise ship named Diamond Princess departed from Yokohama on 20 January. Spread of infection was confirmed on February 4 within the cruise. 218 people onboard tested positive for the virus. In Japan, 3600 people were quarantined in cruise ship. The US passengers of Diamond Princess cruise ship went home for further quarantine by February 17. On February 27, Japan Prime Minister recommended schools to break in order to reduce the spread of coronavirus. By March 5, 98.8% of public elementary schools were closed. Government announced not to continue school closures on March 20. On February 17, the Japanese government also announced plans to expand the national health insurance system so that it covers COVID-19 tests. Months after the first confirmed cases were reported, on April 7, Japan announced a state of emergency in order to prevent the spread of the coronavirus^39^39Wikipedia (2020). Coronavirus pandemic in Japan[online]. Website https://en.wikipedia.org/wiki/2020_coronavirus_pandemic_in_Japan#January [accessed 13 April 2020].,^40^40Wikipedia (2020). Coronavirus pandemic on Diamond Princess [online]. Website https://en.wikipedia.org/wiki/2020_coronavirus_ pandemic_on_Diamond_Princess [accessed 13 April 2020]..

## 5. Education authorities

### 5.1. Schools 

**Figure 1:**
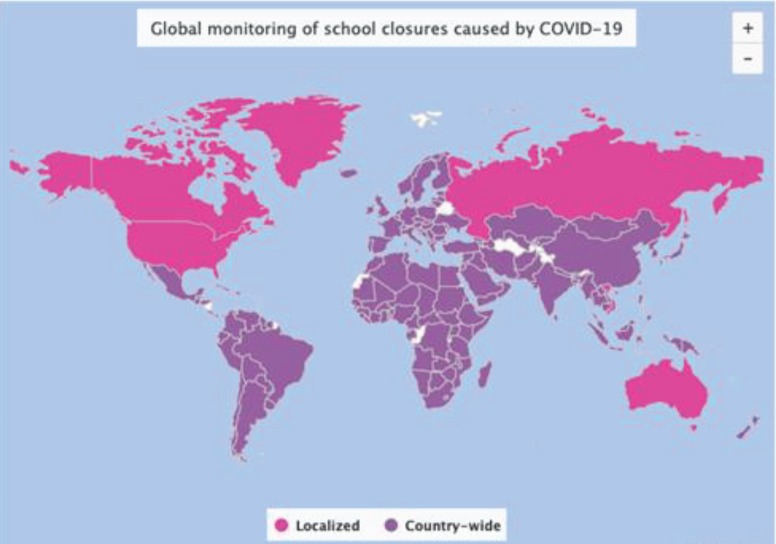
Global monitoring of school closures caused by COVID-19, 188 country-wide closures [38].

The COVID-19 pandemic also effected the continuing education and education institutions. Primary and secondary schools were temporarily closed in most countries, so do the higher education institutions and medical schools. According to UNESCO, the 91% of world’s student population was affected from these nationwide closures. There were also countries in them localized closures were implemented. The more vulnerable and disadvantaged communities were effected more from outbreak because they could not continue with remote or distance learning (Figure 1)^41^41UNESCO (2020). COVID-19 Educational Disruption and Response [online]. Website https://en.unesco.org/covid19/educationresponse [accessed 13 April 2020]..

Distance learning opportunities have never been so much popular worldwide before. A new era begins with COVID-19 pandemic. For a long time, we will be witnessing a wider role of online learning, and massive online open courses (MOOC) education. Teachers are no longer in the schools, students are no longer in the classrooms. The rapid change in learning environment will bring many pro-con discussions for the future. Distance learning does not affect just only students, teachers and school administrators but also parents. Also, resources to provide psychosocial support exist (InterAgency Standing Committee guidelines, WHO mental health and psychosocial guidance and UNICEF guidance). Designing home school, home office, home classrooms, all exaggerated with the pandemic. Distance learning solutions supposed by UNESCO are provided under many titles (Table 4)^42^42UNESCO (2020). Distance learning solutions[online]. Website https://en.unesco.org/covid19/educationresponse/solutions [accessed 13 April 2020]..

**Table 4:** Distance learning solutions supposed by UNESCO in a global pandemic[39].

Digital learning management systems, in different languages · CenturyTech · ClassDojo · Edmodo · Edraak · EkStep · Google Classroom · Moodle · Nafham · Paper Airplanes · Schoology · Seesaw · Skooler	Self-directed learning contents · British Council · Yuj’s · Code It · Code.org · Code Week · Discovery Education · Duolingo · Feed the Monster · Geekie · Khan Academy · KitKit School · LabXchange · Mindspark · Mosoteach · OneCourse · Polyup · Quizlet · Siyavula · Youtube
Systems built for use on basic mobile phones · Cell-Ed · Eneza Education · Funzi · KaiOS · Ubongo · Ustad Mobile	Systems with strong offline functionality · Kolibri · Rumie Ustad Mobile
Massive open online course platforms · Alison · Canvas Network · Coursera · European Schoolnet Academy · EdX · Icourses · Future Learn	Mobile reading applications · African Storybook · Biblioteca Digital del Instituto Latinoamericano de la Comunicación Educativa · Global Digital Library · Room to Read · StoryWeaver · Worldreader

Distance learning cannot be a direct alternative to formal education. It is still important for staying connected to students. There are already many advantages and disadvantages, but as the situation makes it obligatory, many models are on the way and new experiences will form in this pandemic period. According to UNESCO sources, some of the national learning platforms and tools of different countries in the current pandemic are as follows^43^43UNESCO (2020). National learning platforms and tools [online]. Website https://en.unesco.org/covid19/educationresponse/nationalresponses [accessed 13 April 2020].:

· Turkey: *Remote Educational System – The Ministry of National Education will launch a “remote educational system” free of charge on March 23, 2020 with a television and internet-based curriculum on a national scale. Some online resources and applications are already available on the Ministry’s website*.

· Kyrgzystan: *Ibilim – An open educational portal endorsed by the Ministry of Education of Kyrgyzstan with free access to online video and audio lessons for primary school students on mathematics, Kyrgyz, Russian, English languages, music and arts.*

· France: *Ma classe à la maison – The Centre national d’enseignement à distance provides a virtual classroom system accessible via smartphones and computers, enabling teachers to facilitate the organization of distance learning.*

· Italy: *INDIRE webinars – National Institute for Documentation, Innovation and Educational Research [INDIRE] platform providing webinars for teachers to support them in the adoption of distance learning methodologies and tools. Nuovo Coronavirus webpage – The webpage of the Ministry of Education and Higher Education providing information and guidance for education practitioners and families on the education response to COVID-19.*

· Japan: *Future Classroom – A collection of platforms pointing users to a variety of useful sites for teaching and learning.MEXT – Platform to support e-learning by age, level of education and subject.*

· China: *National Cloud – Platform for Educational Resources and Public Service – Provides free teaching and learning resources for primary and secondary school students.*

### 5.2. Universities

COVID-19 pandemic also affected universities. Hundreds of universities were also closed in most countries^44^44UNSECO (2020). National learning platforms and tools [online]. Website https://en.unesco.org/covid19/educationresponse/nationalresponses [accessed 13 April 2020].. Research laboratories suspended their researches as well as the postgraduate studies. In 2020, International Higher Education Forum, there have been rumors that COVID-19 will cause profound impacts and changes on the higher education system around the world in terms of education-teaching methods, researches, internationalization and mobility. Because of the current outbreak, digital education started to become widespread. The social structure will be able to change in the postpandemic era, too. Many students may prefer online education and distance learning, so will many program directors. But, how it will be then. Schools and universities are not just places for lectures, but the interaction of different people, learning respect to each other and human values, communicating in different circumstances etc. Will the world run to an era that not embraces human values? We will see.

CDC is encouraging limiting events and meetings that require close contact if there is no outbreak and cancel large meetings or events during an outbreak and also plan for distance and digital learning in the universities. They also advice monitoring absenteeism, and assess ways to increase physical space between students and limit interactions^45^45CDC (2020). Coronavirus (COVİD-19) [online]. Website u2a06 [accessed 14 April 2020].. Many universities are closed their campuses to students and went through digital learning in COVID-19 outbreak but still some allow researchers to go on. In many countries, universities are autonomous, so in Turkey. They are not under Ministry of Education nor a Ministry of Higher Education exist, but there is the Council of Higher Education [CoHE, YÖK] which is the constitutionally governing body responsible for strategic planning, coordinating, supervising and monitoring of universities in Turkey. The CoHE has been following the outbreak from the first time on and closely monitor roadmaps of official authorities and successful universities of the designated countries in Europe, America and the Far East. When the outbreak did not reach to Turkey yet, measures to be taken in Higher Education Institutions about COVID-19 were released under three titles, which are focusing on infection prevention measures while attending national and international meetings and also avoiding stigma, on 6th of March 2020 (Table 5)^46^46YÖK (2020). Measures to be Taken in Higher Education Institutions about COVID-19 [online]. Website u2a08 [accessed 14 April 2020]..

**Table 5:** Measures to be taken in Higher Education Institutions of Turkey about COVID-19, when there were no reported COVID-19 cases [March 6, 2020] [42].

1. Travel and international meetingsAll of students, academic and administrative staff were advised to reconsider their travel plans, especially their overseas travel plans for reasons such as exchange programs, congresses and meetings and to cancel them in case they were not essential. They were advised to do the following if they were required to travel: a. To check if there were any travel warnings or bans for the country concerned, b. To pay attention to personal hygiene during their travels, c. To visit the nearest health institution if any signs of infection appear during or after their travels and to inform the Turkish Embassy if they were abroad during this time.
2. Meetings with international participation If a high number of attendance was expected from the countries with epidemics in meetings with international participation to be held in our country, it was advised to postpone the planned meeting and consider having online meetings. The following precautions were advised for ongoing meetings; - To provide the necessary environment for hand hygiene and to place hand disinfectants in accessible areas, - To inform and remind the participants about the modes of transmission and prevention methods for COVID-19 before and after the meetings.
3. Measures to be taken against discrimination and stigma- Students and academics from countries with COVID-19, such as China, South Korea, Iran, and Italy, who did not travel to their home countries recently and who any cases of COVID-19 were not reported from their country, were not more prone to contract, carry or transmit the disease, similar to Turkish or other international students. - Utmost attention must be paid to ensure that fear and anxiety about the disease do not cause panic, that people from a certain community or nation are not considered as the source and carrier of the disease, and that international students and academics, especially students and academics from China and other Asian countries, do not face discrimination. - It is essential for the Turkish Academia to be sensitive about this issue and to provide the necessary information and take precautions to prevent mistreatment. - In addition to paying attention to hygiene at campuses, university administrators are advised to hang posters about personal hygiene rules, especially in places such as lecture halls, corridors, dining halls, etc. where people gather, and to share information leaflets with university departments.- Follow the updates on the disease on the official website of the Ministry of Health.

When the first cases of COVID-19 reported in Turkey, immediately the rectors of the universities gathered together in CoHE to discuss the action plan. Around 128 universities in Turkey already had a distance learning center. On March 13, schools are closed nationwide. But only the academicians at risk groups were permitted to stay at home, others supposed to continue their studies to prepare for distance learning in the universities. In an immediate questionnaire 93% said that they can continue with remote or distance learning, 71% with their own base and 22% said they also have to use others infrastructure. In all Turkish universities, New Coronavirus Outbreak Advisory Committee’s (COV COMs) are established to run dynamic solutions for the distance and remote learning and keep the campuses safe and healthy for the remaining staff. Some of immediate actions taken for higher education in COVID-19 pandemic days in Turkey are listed below^47^47YÖK (2020). Press Release -President of The Council of Higher Education [online]. Website u2a0a [accessed 10 April 2020].:

· Continuous and direct connections were established between Higher Education Board Members and university rectorships under the Presidency of Higher Education Council.

· Digital facilities and distance education methods will be used in the theoretical courses of application-based programs and applied courses will be given at the most appropriate time, including the extension of the schedule determined by universities.

· Transition to online education has been initiated. 

· The universities whose Learning Management System [LMS] does not exist or sufficient yet were directed to the universities experienced in this field and their infrastructure was strengthened. 

· “CoHE Courses Programs (yokdersleri.yok.gov.tr) was created. These resources are combination of entertainment and academic material

· The enhancement recommendations were submitted concerning the staff and personal rights of healthcare professionals working in university hospitals, which are important for health education, to the government, and these enhancements were supported, and necessary arrangements were made.

· The students are allowed to suspend their studies or postpone their enrollment

Although the universities can use distance learning methods synchronously or asynchronously in all courses in formal education programs, the practices for different programs and clinical practices for the medical school students are concerning. About the students’ and trainees’ involvement in the care of COVID-19 patients are important issues to be solved for different universities. In USA, the high probability that medical students in the hospital would be exposed to outbreak cases and the need to conserve PPE seemed to outweigh the educational benefits of students’ participation. The Association of American Medical Colleges recommended that member schools suspend clinical rotations for medical students for several weeks [6,7]. In Turkey, although all formal education in campuses are suspended in the universities, the medical schools are allowed to make their own decision about last year medical students to continue or not to their clinical practices. Also voluntarily work within the hospitals are allowed for those last year students who were nearly to graduate after completing their internships^48^48YÖK (2020). Press Release -President of The Council of Higher Education [online]. Website https://www.yok.gov.tr/en/Sayfalar/ news/2020/Sarac-made-a-statement-on-the-distance-education.aspx [accessed 10 April 2020]..

Different examples from different countries exist. Although Harvard University designs many thinks to be remote and distant (teach remotely, learn remotely, work remotely, research remotely, socialize remotely) anymore, they underline that medical students need to complete rotations and patient exams to meet graduation requirements, but, of course, with ensuring the safety of students, patients, staff and faculty. They also recommend medical students not be involved in the care of patients with confirmed or suspected COVID-19^49^49Harvard University (2020). Coronavirus (COVİD-19) [online]. Website https://www.harvard.edu/coronavirus [accessed 10 April 2020].. 

The universities and other higher education institutions play extremely important role because they can help to slow the spread of the outbreak; improve guidelines; save the society and keep them in a physiologically safe mood; publish papers; run webinars, work with local health departments, invent new tools for the diagnosis; and make research on the virus and potential vaccines etc. The national authorities also provide sources with the help of academicians and for helping the academicians. As an example, besides funding researches, The Turkish Scientific and Technological Research Council of Turkey (TÜBİTAK) opened a portal about COVID-19 which is very useful for being update in a pandemic.

## 6. Conclusion

An outbreak can emerge all of a sudden or sometimes there may be signs of it beforehand. Active surveillance, infection control measures are extremely important but may be not enough all the times. Usually there are local guidelines for institutional roles in case of an outbreak, mainly guided by local health and governor authorities. In case of a pandemic, controlling the outbreak is a much bigger problem and difficult issue. In a pandemic, there are no borders anymore. Each country could be affected from others’ decisions and infection control measures. The world population will be under risk of similar agent, regardless of their citizenships, countries or welt. All national and international institutions should be connected with each other, work together, share experience, publish guideline for general population, heath care facilities, local authorities, public service facilities, students etc. In Turkey’s case, the Ministry of Health, the Council of Higher Education, the Ministry of Education and all other institutions response to the current pandemic can be a model for future studies, hopefully continuously after the end of pandemic. The COVID-19 Advisory Committee, which consists of professionals from different areas was extremely important to monitor the outbreak and provide advices after evaluating updated information.

Organizations like WHO and UNESCO are extremely important to keep the countries updated, share different countries’ measures and show the global aspect. They have to be clear and transparent in each step not to lost the trust of the populations. Besides their informative roles managing bridges between different countries and providing the important supplies like PPE and hygiene products to those countries in need are extremely important roles in a pandemic. Health protection agencies and CDC like institutions have leader roles guiding the authorities about the virus’ characteristics, reading the outbreak curve, measures to be taken etc. Health ministries of countries should guide the operational plans in different levels. Other ministries should follow the advices and proposals to manage their routine work in a pandemic era. Educational institutions, primary and secondary schools should continue to connect with their students not to lost even a year for a generation in a pandemic. Universities should not suspend their important researches especially those which can help for prevention and control of the pandemic, but try to continue as much as the outbreak allows. 

## Acknowledgments/disclaimers

Zeliha KOÇAK TUFAN is an executive board member of CoHE of Turkey, board member of COVID-19 Advisory Committee of the MoH of Turkey, has been in the Influenza Scientific Board of MoH for years. Bircan KAYAASLAN is working in the main pandemic hospital, Ankara City Hospital, a hospital with 3810 total hospital beds and 696 ICU beds.
